# Surgical outcomes of Ahmed Glaucoma Valve Implantation surgery: With mitomycin C and without mitomycin C

**DOI:** 10.4102/jcmsa.v4i1.362

**Published:** 2026-05-26

**Authors:** Hojatollah Jaldi, Tshilidzi van der Lecq

**Affiliations:** 1 Division of Ophthalmology, Department of Surgery, Faculty of Health Sciences, University of Cape Town, Cape Town, South Africa

**Keywords:** glaucoma, intraocular pressure, Ahmed Glaucoma Valve, Mitomycin C, surgical outcomes, African, glaucoma drainage devices, postoperative outcomes

## Abstract

**Background:**

The Ahmed Glaucoma Valve (AGV) is a commonly used glaucoma drainage device (GDD) in the surgical management of glaucoma patients. Adjunctive mitomycin C (MMC) has demonstrated clear benefits in improving the surgical outcomes of trabeculectomy surgery. The effectiveness of MMC in improving the surgical outcomes of GDDs remains uncertain. This study seeks to compare the surgical outcomes of AGV implantation with and without the use of intraoperative MMC in an African cohort.

**Methods:**

This retrospective case series analysed 98 eyes in 95 patients who underwent AGV implantation from 01 January 2016 to 31 December 2021 at two units located in Cape Town, South Africa. The postoperative data collected included intraocular pressure (IOP), medication use, and complications.

**Results:**

The eyes were categorised into the MMC use (*n* = 46) or non-MMC use (*n* = 52) group. In most eyes within the MMC use group (78.3%, *n* = 36), a concentration of 0.2 mg/mL was used. The mean preoperative IOP in the MMC group and the non-MMC group was 38 and 33, respectively (*p* = 0.12). At 1 year, the mean IOP in the MMC use group (*n* = 31) was 18 mmHg and 16 mmHg in the non-MMC group (*n* = 36) (*p* = 0.84). The frequency of complications and the hypertensive phase were comparable in the two groups.

**Conclusion:**

This study found no difference between the two groups in terms of IOP control, postoperative medication use, and complications. This may be attributed to the low concentration of MMC used in our cohort.

**Contribution:**

This study provides insights into the role of MMC use in AGV implantation.

## Introduction

Glaucoma is the leading cause of irreversible blindness worldwide,^1^ and lowering intraocular pressure (IOP) remains the only proven method to slow its progression.^2^ While trabeculectomy surgery has traditionally been the gold-standard surgical procedure for reducing IOP, the use of glaucoma drainage devices (GDDs) has increased in recent years.^3^ These devices offer advantages such as a simpler surgical technique and fewer postoperative complications.^4,5^ Initially reserved for refractory cases, these devices are now increasingly used as primary surgical interventions.^3,5,6,7^ Among these devices, the Ahmed glaucoma valve (AGV) is one of the most widely used.^5,6^

Mitomycin C (MMC), an anti-fibrotic agent, is commonly used as an adjunct in glaucoma filtration surgeries.^7^ It inhibits fibroblast proliferation and scar formation, thereby enhancing aqueous outflow, reducing IOP, and improving surgical outcomes.^6,8^ While the benefits of MMC in trabeculectomy surgery are well documented,^5,9,10^ its role in GDD surgeries remains controversial.^1,9^

One of the disadvantages of AGV implantation is the high incidence of the hypertensive phase, defined as an IOP > 21 mmHg occurring during the first 3 months after surgery. This is associated with an increased risk of long-term surgical failure.^5,11^ Some studies have reported that intraoperative MMC lowers postoperative IOP and reduces the incidence of the hypertensive phase.^9,11,12,13,14,15,16,17^ Several authors have confirmed the finding that MMC use reduces the postoperative IOP^5,14^ and can reduce antiglaucoma medication reliance up to 1 year after surgery.^5^

However, some authors found that while MMC initially reduced IOP after AGV implantation, this reduction was not sustained in the long term. In studies by Kim et al. and Yazdani et al., MMC use resulted in a lower IOP 1 month after surgery, but was comparable to cases without MMC use at 6 and 12 months.^8,18^ One hypothesis is that the antiproliferative effects of MMC during the initial wound-healing phase are overwhelmed by a persistent immune response and fibroblastic proliferation elicited by the drainage device.^19^ This theory may explain the differences in long-term outcomes between MMC use in trabeculectomy surgery and GDDs.^8,10^

In terms of safety, complication rates in eyes with MMC use versus those without MMC use are either comparable or show no statistically significant difference.^5,8,10,17,18,19^ Three studies suggested the increased risk of postoperative hypotony among eyes with MMC use, although not a statistically significant finding.^12,16,20^

Importantly, studies assessing the use of MMC have used a wide range of concentrations (0.2 mg/mL – 0.5 mg/mL) applied for different durations (2–8 min). In a recent randomised clinical trial, Swampillai et al. used a MMC dose of 0.4 mg/mL for 3 min in African and African-Caribbean patients. The authors demonstrated favourable outcomes, including a lower mean IOP and a higher proportion of patients achieving medication-free status at 12 months.^7^ The concentration and duration of MMC use may influence postoperative outcomes.

African patients undergoing glaucoma filtration surgery have a higher risk of surgical failure because of increased postoperative scarring.^7,21^ Data regarding the benefit of MMC use in this population could help to improve GDD-related surgical outcomes. The aim of this study was to compare the surgical outcomes in eyes undergoing AGV implantation with and without intraoperative MMC use.

## Research methods and design

This study was a retrospective case series of eyes receiving AGV implantation with or without the use of intraoperative MMC at Groote Schuur Hospital and Eerste River Hospital in Cape Town, South Africa. All consecutive patients who received primary AGV implantation between 01 January 2016 and 31 December 2021 were included. Patients with incomplete medical records or a history of previous GDD surgery were excluded. Eyes were categorised into those that received intraoperative MMC (MMC group) and those that did not (non-MMC group).

### Data collection

Medical records were reviewed to extract the data of those eyes eligible for inclusion. Preoperative, intraoperative, and postoperative data were captured with a postoperative follow-up period of up to 3 years (extending up to 31 August 2024), depending on data availability. The variables captured included: demographic details, preoperative and postoperative IOP, indication for surgery, preoperative procedures, preoperative and postoperative antiglaucoma medications, diagnosis of the hypertensive phase, timing at which medications were introduced postoperatively and postoperative complications. Maximum medical therapy was defined as the use of four antiglaucoma agents.

### Surgical technique

Ahmed glaucoma valve (New World Medical, Rancho Cucamonga, California) implantation was performed using a well-described, standardised technique, briefly summarised next.^22^ Surgeries were performed by qualified ophthalmologists or supervised ophthalmology residents. General or local (sub-tenon block) anaesthesia was used as indicated. Placement of a corneal traction suture was followed by conjunctival peritomy, blunt dissection and haemostasis via cautery. In the MMC group, MMC at a concentration of 0.2 mg/mL or 0.4 mg/mL was applied to the subconjunctival or sub-tenon space for 2–4 min, based on the surgeon’s preference. The AGV was primed, positioned at least 8 mm behind the limbus and secured using 8-0 Prolene or 6-0 Mersilene sutures. The tube was most commonly inserted into the anterior chamber and, in some cases, placed in the ciliary sulcus or pars plana. A scleral patch graft was placed over the tube and the conjunctival flap secured using interrupted 10-0 nylon sutures. At the end of the procedure, intracameral moxifloxacin (0.5 mg/0.1 mL) was injected into the anterior chamber. In some instances, 1.25 mg of bevacizumab (Avastin, Genentech, United States) was also injected into the anterior chamber based on the surgeon’s preference.

### Data analysis

Data were analysed using IBM SPSS version 28 (IBM Inc., Chicago, Illinois, United States). Continuous variables were summarised using means and standard deviations, while categorical variables were presented as frequencies and percentages. Independent *t*-tests and Chi-square tests were used to assess differences between the MMC and non-MMC group. Statistical significance was set at *p* < 0.05.

### Ethical considerations

The study adhered to the principles of the Declaration of Helsinki, and ethical approval was obtained from the University of Cape Town Human Research Ethics Committee (HREC reference no. 867/2023).

## Results

### Baseline characteristics

The study included 98 eyes of 95 patients, with 46 eyes in the MMC group and 52 eyes in the non-MMC group. Within the total cohort, 50 (51.0%) patients were female, and the mean age was 57 (range 44–64) years (Table 1). Neovascular glaucoma (NVG) was the most common indication for AGV implantation in 42 (42.9%) eyes. Most eyes, 85 (86.7%), were on maximum medical therapy prior to surgery and 75 eyes (76.5%) had undergone prior surgical procedures, mainly phacoemulsification with intraocular lens implantation 39 (39.8%) (Table 1).

**TABLE 1:** Baseline characteristics of mitomycin C and non-mitomycin C groups.

Category	All eyes		MMC		Non-MMC		*p*-value†
	*n*	%	*n*	%	*n*	%	
**Sex**							
Male	48	49.0	26	56.5	22	42.3	0.30
Female	50	51.0	20	43.5	30	57.7	-
Total	98	-	46	47.0	52	53.0	-
Maximum preoperative medical therapy‡	85	86.7	37	80.4	48	92.3	0.08
**Preoperative glaucoma diagnosis**							
Primary open-angle glaucoma (POAG)	15	15.3	8	17.4	7	13.5	0.60
Neovascular glaucoma	42	42.9	23	50.0	19	36.5	0.18
Juvenile open-angle glaucoma	2	2.0	0	0.0	2	3.8	0.18
Uveitic glaucoma	17	17.3	9	19.6	8	15.4	0.58
Closed-angle glaucoma	5	5.1	2	4.3	3	5.8	0.75
Post-retinal surgery	4	4.1	1	2.2	3	5.8	0.37
Secondary open-angle glaucoma	8	8.2	1	2.2	7	13.5	0.04
Other§	5	5.1	2	4.3	3	5.8	0.75
**Preoperative procedures or surgery**							
None	20	20.4	8	17.8	12	23.1	0.01
Trabeculectomy	18	18.4	5	11.1	13	25.0	0.10
Cataract surgery	39	39.8	21	45.6	18	34.6	0.15
Pars plana vitrectomy	11	11.2	4	8.9	7	13.5	0.54
Laser photocoagulation	20	20.4	12	26.1	8	15.4	0.13
Intravitreal anti-VEGF	28	28.6	18	39.1	10	19.2	0.02
Selective laser trabeculoplasty	3	3.1	3	6.7	0	0.0	0.05
Yag peripheral iridotomy	2	2.0	1	2.2	1	1.9	0.89
Missing values	2	2.0	1	2.2	1	1.8	-

VEGF, vascular endothelial growth factor; MMC, mitomycin C group; non-MMC group, non-mitomycin C group.

† , *p*-value compares the MMC and the non-MMC group;

‡ , maximum therapy indicates the use of four antiglaucoma agents;

§ , includes iridocorneal endothelial glaucoma, congenital glaucoma, and aphakic glaucoma.

### Intraocular pressure reduction

**FIGURE 1:**
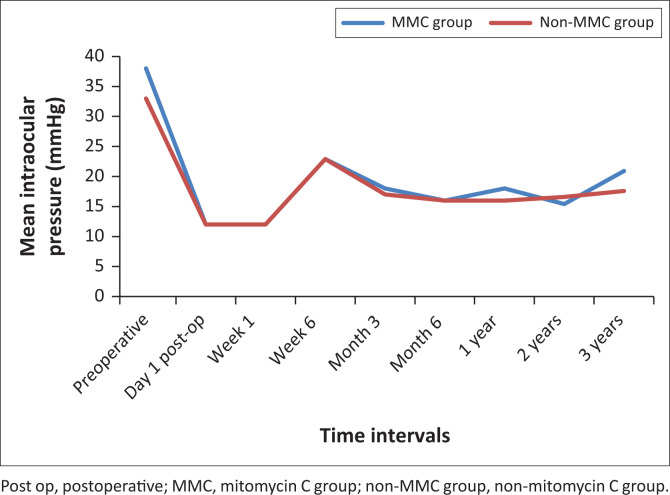
Mean intraocular pressure at different time intervals in mitomycin C and non-mitomycin C groups.

The preoperative mean IOP in the entire cohort was 36 mmHg (range: 26 mmHg – 47 mmHg), and it was comparable between the MMC and non-MMC groups: 38 mmHg (range: 30 mmHg – 49 mmHg) and 33 mmHg (range: 24 mmHg – 42 mmHg), respectively (*p* = 0.12) (Figure 1). In the MMC group, 78.3% (*n* = 36) of eyes received MMC at a concentration of 0.2 mg/mL, and 80.4% (*n* = 37) had it applied for 2 min.

The mean IOP within the total cohort at 1 year, 2 years, and 3 years was 17 (standard deviation [s.d.] 6.7) mmHg, 16 (s.d. 6.6) mmHg, and 19 (s.d. 7.4) mmHg, respectively. At the end of the first postoperative year, the mean IOP in the MMC group (*n* = 31) was 18 mmHg (s.d. 6.3), while in the non-MMC group (*n* = 36) it was 16 (s.d. 7.0) mmHg (*p* = 0.84). At the end of the second year, the mean IOP in the MMC group (*n* = 24) was 15 (s.d. 5.4) mmHg, compared to 17 (s.d. 7.3) mmHg in the non-MMC group (*n* = 33) (*p* = 0.47). By the end of the third year, the mean IOP in the MMC group (*n* = 13) was 21 (s.d. 6.0) mmHg, and in the non-MMC group (*n* = 22) it was 18 (s.d. 8.1) mmHg (*p* = 0.17).

### Antiglaucoma medication use

**FIGURE 2:**
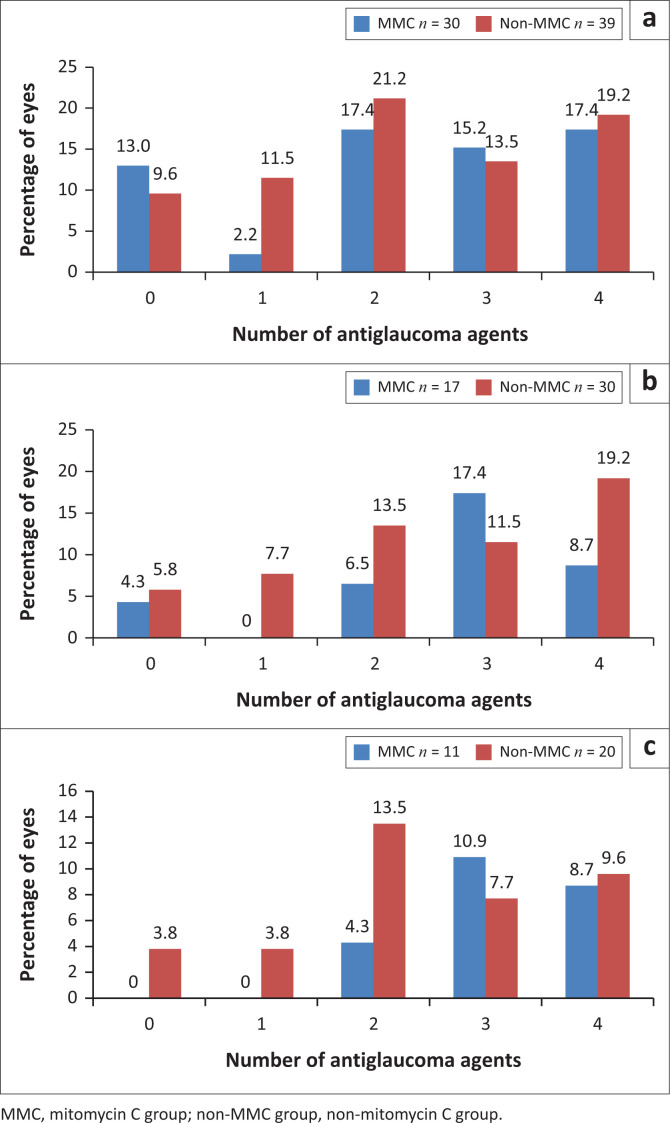
Antiglaucoma use in mitomycin C and non-mitomycin C groups at (a) 1 year, (b) 2 years and (c) 3 years.

Preoperatively, in the MMC group, 37 eyes (80.4%) were on maximum medical therapy (i.e. four agents) compared to 48 eyes (92.3%) in the non-MMC group. Figure 2a–c illustrate the number of agents required in both groups at the end of the first, second, and third postoperative year, respectively.

### Hypertensive phase characteristics

In the total cohort, 68 (69.4%) eyes developed the hypertensive phase. This represented 31 eyes (67.4%) in the MMC group and 37 eyes (71.2%) in the non-MMC group.

**FIGURE 3:**
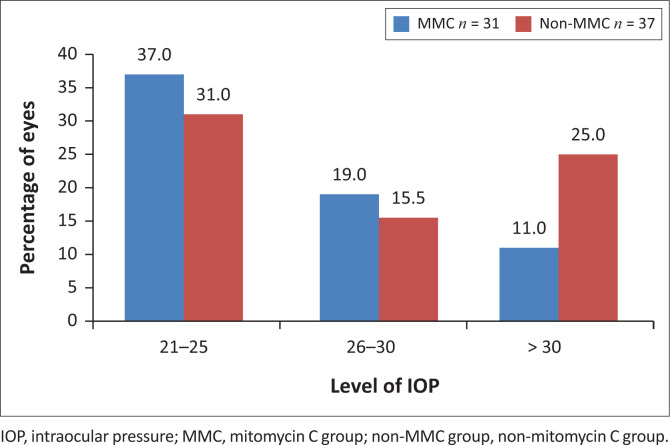
Level of intraocular pressure at diagnosis of the hypertensive phase (*n* = 68).

The IOP level at the diagnosis of the hypertensive phase in the 68 eyes is illustrated in Figure 3. The IOP levels within the MMC group and the non-MMC group were as follows: 21 mmHg – 25 mmHg (17 [36.9%] eyes vs. 16 [30.8%] eyes), 26 mmHg – 30 mmHg (9 [19.5%] eyes vs. 8 [15.4%] eyes), and IOP > 30 mmHg (5 [10.8%] eyes vs. 13 [25%] eyes).

### Postoperative complications

Overall, 54 eyes (55.1%) developed a postoperative complication after AGV implantation, 20 (43.5%) eyes in the MMC group and 34 (65.4%) in the non-MMC group (*p* = 0.04). In the total cohort of eyes, the most common postoperative complication was an encysted bleb in 16 eyes (16.3%) (Table 2). This affected more eyes in the non-MMC group than in the MMC group: 12 (23.1%) and 4 (8.7%), respectively, *p* = 0.06. Hypotony occurred less frequently in the non-MMC group compared to the MMC group (3.8% vs. 6.5%), *p* = 0.55. Complications such as membrane growth over the tube, blebitis and endophthalmitis were not observed in either group.

**TABLE 2:** Distribution of postoperative complications in the mitomycin C and non-mitomycin C groups.

Complication	Total cohort (*n* = 98)		MMC group (*n* = 46)		Non-MMC group (*n* = 52)		*p*-value†
	*n*	%	*n*	%	*n*	%	
Encysted bleb	16	16.3	4	8.7	12	23.1	0.06
Hypotony	5	5.1	3	6.5	2	3.8	0.66
Tube erosion	4	4.1	2	4.3	2	3.8	1.00
Tube migration	1	1.0	0	0.0	1	1.9	1.00
Tube cornea touch	1	1.0	1	2.2	0	0.0	0.47
Suprachoroidal haemorrhage	2	2.0	0	0.0	2	3.8	0.50
Corneal decompensation	3	3.1	0	0.0	3	5.8	0.24
Ongoing proliferation resulting in vitreous haemorrhage	8	8.2	3	6.5	5	9.6	0.72
Occluded tube	5	5.1	2	4.3	3	5.8	1.00
Cataract	1	1.0	1	2.2	0	0.0	0.49

MMC, mitomycin C; non-MMC, non-mitomycin C.

† , *p*-value compares the MMC and the non-MMC group.

## Discussion

This study compared the surgical outcomes in eyes undergoing AGV implantation with and without intraoperative MMC use. This study found no difference in postoperative IOP reduction, the frequency of the hypertensive phase, or the need for postoperative medication use between the two groups.

In our study, the postoperative IOP reduction at 1 year was similar in the MMC and non-MMC groups. Our results are consistent with several previous studies that also reported no significant improvement in IOP control at 1 year with intraoperative MMC use.^8,19,20^

However, Mahdy et al. and Tien et al. found that eyes treated with MMC had significantly larger reductions in postoperative IOP. In Mahdy et al.’s study, the mean IOP in the MMC group dropped from 32 mmHg preoperatively to 14.0 mmHg at 1 year, compared to a reduction from 35 mmHg to 17.0 mmHg in the non-MMC group (*p* < 0.05). Additionally, Tien et al. reported a statistically significant difference in surgical success rates (*p* = 0.01), with the MMC-treated group being 4.86 times more likely to achieve IOP success compared to those who did not receive MMC. This difference may be because of the higher concentration of MMC used or variations in the method of MMC application in their studies compared to this study.^10,14,17^

Fibrous encapsulation of the AGV plate is a well-recognised contributor to postoperative IOP elevation and long-term surgical failure.^9,19^ Mitomycin C, an antimetabolite, is often used to mitigate this fibrotic response.^8,10^ Although not significant, our findings suggest a trend (*p* = 0.06) between MMC use and fewer encysted blebs that is consistent with findings by Zhou et al.^15^ This indicates that MMC can favourably modulate wound healing, even in the absence of long-term IOP benefit.

In our cohort, the frequency of complications appeared to be higher in the non-MMC group (65.4%, *n* = 34/52) than in the MMC group (43.5%, *n* = 20/46) (*p* = 0.04). This higher complication rate in the non-MMC group may be partly attributable to the greater incidence of encysted blebs (23.1% vs. 8.7%) (*p* = 0.06). A transient hypotony occurred with the same frequency in the MMC group and the non-MMC group (6.5% vs. 3.8%, *p* = 0.66), similar to findings by Kurnaz et al.^20^ Various mechanisms responsible for hypotony with AGV implantation include: peritubular leakage, reduced aqueous production by the ciliary body, and failure of the valve mechanism.^4^ Importantly, serious complications such as blebitis or endophthalmitis did not occur in either of the groups. This is in keeping with the findings of Peixoto et al. and Kook et al., who reported comparable safety profiles even with higher MMC concentrations.^12,16^ However, the use of high-dose MMC (i.e. 0.4 mg/mL for 5 min) has been associated with serious complications such as scleromalacia.^22^

The heterogeneity in MMC use protocols is a key challenge in interpreting and comparing results across studies. There can be differences in concentration (ranging from 0.2mg/mL to 0.5 mg/mL), duration of application (2–8 min), and delivery method (e.g. subconjunctival injection vs. sponge application).^5,6,7,8,9,20^ Such variability may account for inconsistencies in the reported postoperative outcomes. For example, Mahdy et al. and Kook et al. reported improved outcomes using 0.4 mg/mL for 3–5 min, with reduced IOP and medication burden at 1 year.^14,16^ In contrast to these studies, about 80% of eyes in our cohort received a relatively low concentration (0.2 mg/mL) of MMC, applied for only 2 min. These parameters may be suboptimal for achieving MMC’s maximal antifibrotic effect. The lower MMC dosage and shorter exposure time in our study could account for the comparable surgical outcomes between the two groups. Supporting this, a recent randomised trial by Swampillai et al. in African and African-Caribbean patients used a 0.4 mg/mL MMC concentration applied for 3 min. They found that the MMC group had a lower mean IOP at 1 year (13 mmHg vs. 15 mmHg, *p* = 0.27), although this difference was not statistically significant. However, there was a statistically significant difference in the mean number of medications used in the two groups (*p* = 0.005) at 1 year.^7^

### Limitations

Our study had some limitations. The retrospective design of the study and reliance on medical records may have introduced selection and information bias. The unmeasured use of adjunctive therapies in some cases (e.g. anti-vascular endothelial growth factor agents) could have influenced postoperative outcomes between the two groups. Additionally, the coronavirus disease 2019 pandemic interrupted routine follow-up visits, compromising the completeness of the long-term data. Lastly, NVG, a well-known risk factor for GDD failure,^23^ accounted for almost half of the cases in this cohort, and may have skewed the overall results and comparison between the two groups.

### Strengths

Compared to prior studies, the relatively large cohort size, standardised surgical technique, and 3-year follow-up in some cases are key strengths of this study.

## Conclusion

This study found no significant differences between the MMC and non-MMC groups in IOP control, postoperative medication use, or the incidence of the hypertensive phase and complications. These outcomes may reflect the low concentration of MMC used in our cohort. Given the higher risk of surgical failure in African glaucoma patients because of excessive scarring, further studies are needed. Prospective randomised studies comparing higher concentrations of MMC applied for longer durations may reveal improved surgical outcomes in AGV implantation in this cohort.
